# Rhizobacteria promote plant growth via secretion of N-(3-oxooctanoyl)-L-homoserine lactone

**DOI:** 10.1093/hr/uhag071

**Published:** 2026-02-28

**Authors:** Hongfei Li, Dongxin Liu, Huailong Teng, Lile Deng, Bo Zheng, Qiang Xu, Shunyuan Xiao, Xiuxin Deng, Zhiyong Pan

**Affiliations:** National Key Laboratory for Germplasm Innovation & Utilization of Horticultural Crops, College of Horticulture and Forestry Sciences/Chemistry of Huazhong Agricultural University, Wuhan 430070, China; National Key Laboratory for Germplasm Innovation & Utilization of Horticultural Crops, College of Horticulture and Forestry Sciences/Chemistry of Huazhong Agricultural University, Wuhan 430070, China; National Key Laboratory for Germplasm Innovation & Utilization of Horticultural Crops, College of Horticulture and Forestry Sciences/Chemistry of Huazhong Agricultural University, Wuhan 430070, China; National Key Laboratory for Germplasm Innovation & Utilization of Horticultural Crops, College of Horticulture and Forestry Sciences/Chemistry of Huazhong Agricultural University, Wuhan 430070, China; National Key Laboratory for Germplasm Innovation & Utilization of Horticultural Crops, College of Horticulture and Forestry Sciences/Chemistry of Huazhong Agricultural University, Wuhan 430070, China; National Key Laboratory for Germplasm Innovation & Utilization of Horticultural Crops, College of Horticulture and Forestry Sciences/Chemistry of Huazhong Agricultural University, Wuhan 430070, China; Institute for Bioscience and Biotechnology Research and Department of Plant Sciences and Landscape Architecture, University of Maryland College Park, Rockville, MD 20850, USA; National Key Laboratory for Germplasm Innovation & Utilization of Horticultural Crops, College of Horticulture and Forestry Sciences/Chemistry of Huazhong Agricultural University, Wuhan 430070, China; National Key Laboratory for Germplasm Innovation & Utilization of Horticultural Crops, College of Horticulture and Forestry Sciences/Chemistry of Huazhong Agricultural University, Wuhan 430070, China

## Abstract

Plant growth-promoting rhizobacteria (PGPR) interact with host plants through chemical signals. However, the specific signals in citrus–PGPR interactions remain unclear. Here, we show that a predominant and growth-promoting *Burkholderia* strain (Burk_2H3) isolated from citrus rhizosphere promotes plant growth by secreting N-(3-oxo-octanoyl)-L-homoserine lactone (PGPHL). Metabolomic analysis revealed that PGPHL abundance in Burk_2H3 secretions was 9.7- to 17.2-fold higher than that in three non-promoting *Burkholderia* strains. Exogenous application of PGPHL, but not other secretory metabolites, increased citrus seedling dry weight by 43.12%. Transcriptomic analysis showed that Burk_2H3, its cell-free supernatant, or PGPHL consistently upregulated key nutrient transporter genes in roots. Consistently, ionomic analysis confirmed higher root concentrations of nitrogen, phosphorus, and potassium. Field trials further demonstrated that PGPHL increased biomass by 21% in pepper, 15% in celery, and 18% in mustard. Together, these findings identify PGPHL as a candidate for developing plant growth stimulants and biofertilizers.

## Introduction

The rhizosphere, a dynamic interface shaped by root exudates and microbial activities, harbors diverse rhizobacteria that critically influence plant growth, stress resilience, and soil health [1–3]. Among these microorganisms, plant growth-promoting rhizobacteria (PGPR) enhance host fitness through multifaceted mechanisms [4]. These include the production of bioactive substances such as phytohormones (e.g. Indole-3-acetic acid), 1-aminocyclopropane-1-carboxylic acid (ACC) deaminase, phytase, and siderophores to facilitate nutrient acquisition [5, 6], as well as the suppression of pathogens via antibiotics or lytic enzymes [7].

Secretion of bioactive compounds has emerged as a key strategy by which PGPR modulate plant growth [8, 9]. For instance, auxins and cytokinins promote root architecture and vegetative growth [10, 11]; organic acids, protons, or phytase from strains, such as *Arthrobacter*, *Enterobacter*, and *Pseudomonas* solubilize insoluble phosphates [12, 13]; siderophores from *Bacillus* and *Burkholderia* enhance iron uptake [14, 15]; and glutathione secreted by *Streptomyces* facilitates sulfur assimilation and plant–microbe synergy under nutrient stress [16]. In parallel, many PGPR suppress pathogens by producing antibiotics (e.g. kanosamine, amphotericin A, oligomycin A, flavobactin) and secreting cell wall-degrading enzymes [17, 18]. Despite these advances, current understanding of the specific chemical signals mediating PGPR–plant interactions remains largely derived from herbaceous models. Woody plants, particularly perennial fruit crops, such as citrus, represent a critical research gap in this field.

Citrus, a globally significant fruit crop, faces persistent challenges, such as nutrient deficiencies and pathogen susceptibility, which affect yield sustainability [19, 20]. While harnessing PGPR offers a promising solution, the specific microbial signals and molecular mechanisms mediating beneficial citrus–PGPR interactions remain poorly understood. To address this gap, we isolated *Burkholderia* sp. strain Burk_2H3, a citrus rhizosphere-enriched PGPR, and investigated its growth-promoting effects. Using an untargeted metabolomics approach coupled with functional validation, we identified the key bacterial metabolite plant growth-promoting homoserine lactone (PGPHL) as responsible for the observed growth promotion. We further show that PGPHL enhances citrus growth, and this effect is associated with transcriptional changes in root nutrient uptake pathways. Our findings not only bridge a critical knowledge gap in PGPHL-mediated plant–microbe communication in woody crops, but also provide mechanistic insights into how specific rhizobacterial metabolites can be harnessed to modulate plant performance.

## Results

### A dominant and conserved *Burkholderia* strain as well as its secretions significantly promote plant growth

**Figure 1:**
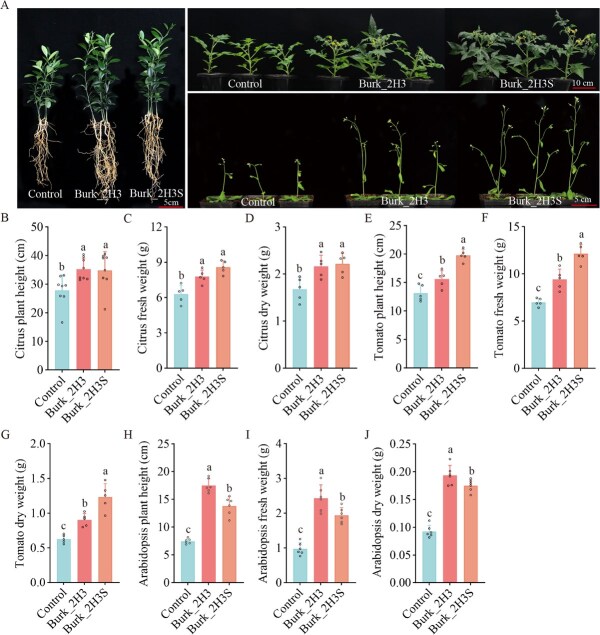
Growth status of potted citrus, tomato and thale cress plants inoculated with Burk_2H3 or Burk_2H3S (A). Plant height (B, E, H), fresh weight (C, F, I) and dry weight (D, G, J) were determined at 6 weeks post-inoculation for citrus and 4 weeks post-cultivation for tomato and thale cress. Data are expressed as mean ± standard deviation of 8 or 6 independent biological replicates (8 citrus plants or 6 tomato and thale cress plants, Tukey's HSD test).

To explore potential PGPR, we initiated a rhizosphere microbiota analysis and revealed that *Burkholderia* sp. strain Burk_2H3 emerged as a predominant rhizobacterial species, with confirmed presence across 15 distinct citrus-growing sites (Fig. S1, Table S1) [21]. Co-culture experiments demonstrated that Burk_2H3 enhanced thale cress (*Arabidopsis thaliana*) biomass and lateral root development even in the absence of direct root contact (Fig. S2A–D), which implies that growth promotion via secreted metabolites. To test this, we compared the effects of Burk_2H3 and its secreted metabolites (Burk_2H3S) across citrus (*Fortunella hindsii*), tomato (*Solanum lycopersicum*, Micro-tom), and thale cress. Both treatments increased citrus growth metrics, including plant height (with additional 25.13% and 28.87%), fresh weight (with additional 23.71% and 36.42%), and dry weight (with additional 26.43% and 32.08%) relative to the controls (Fig. 1A–D), with analogous growth enhancements observed in tomato and thale cress (Fig. 1A, E–J). These findings suggest that Burk_2H3 promotes plant growth primarily through secreted metabolites.

### Burk_2H3 promotes plant growth via secreting PGPHL

**Figure 2:**
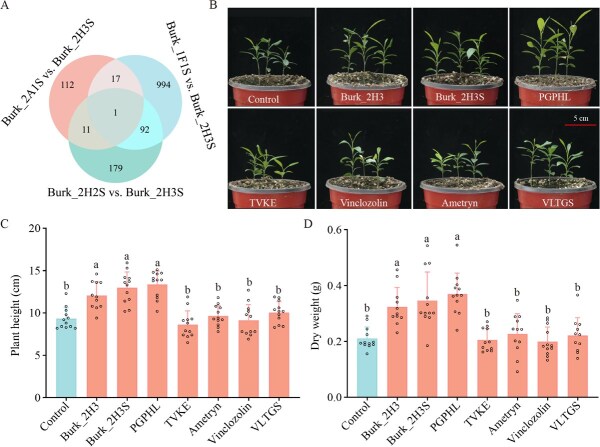
Identification of Burk_2H3S growth-promoting bioactive substance. (A) Venn diagram of differential metabolites in comparisons of Burk_1F1S vs. Burk_2H3S, Burk_2A1S vs. Burk_2H3S, and Burk_2H2S vs. Burk_2H3S. (B) The growth performance of citrus plants co-cultivated with potential bioactive substances. (C) Statistical analysis of plant height of citrus plants. (D) Statistical analysis of dry weight of citrus plants. Data are expressed as mean ± standard deviation of 12 independent biological replicates (Tukey's HSD test).

To identify the secreted bioactive metabolites, we conducted non-targeted metabolomic profiling of Burk_2H3S against the secretions of three non-promotive *Burkholderia* strains (Burk_2A1, Burk_1F1, Burk_2H2) [21]. Among 7473 detected metabolites, differential abundance analysis revealed 1111 (Burk_1F1S vs. Burk_2H3S), 141 (Burk_2A1S vs. Burk_2H3S), and 283 (Burk_2H2S vs. Burk_2H3S) significantly altered metabolites (Fig. 2A, Tables S2–S4). Cross-comparison revealed 18, 12, and 93 overlapping metabolites in pairwise contrasts (Fig. 2A). Among these, N-(3-Oxo-octanoyl)-l-homoserine lactone, a quorum-sensing (QS) signal of the acyl-homoserine lactone (AHL) family, was the sole metabolite universally upregulated in Burk_2H3S across all comparisons.

Functional validation of candidate metabolites revealed that PGPHL uniquely enhanced thale cress lateral root density and primary root elongation (Fig. S3), while significantly boosting height and biomass in citrus (Fig. 2B–D) and tomato (Fig. S4A–C). In contrast, the control substances Val–Leu–Thr–Gly–Ser (VLTGS), Thr–Val–Lys–Glu (TVKE), Ametryn, and Vinclozolin, which were upregulated across both cross-comparisons, showed negligible or inhibitory effects in functional assays (Fig. 2B–D, Fig. S4). Specifically, VLTGS and TVKE were significantly upregulated in Burk_2H3S compared to both Burk_1F1S and Burk_2A1S. Similarly, Ametryn and Vinclozolin showed considerable upregulation in Burk_2H3S compared to both Burk_2H2S and Burk_2A1S. Notably, functional validation of S-adenosylmethionine (SAM), a biosynthetic precursor of PGPHL, confirmed its lack of growth-promoting activity (Fig. S5). This further supports that the observed plant growth promotion is specifically attributed to PGPHL rather than its metabolic precursor. This confirms that N-(3-oxooctanoyl)-L-homoserine lactone, the predominant AHL secreted by Burk_2H3, is the key metabolite responsible for the observed plant growth promotion; it is designated PGPHL.

### Burk_2H3 and its signal PGPHL activate transporter gene expression and promote nutrient uptake

**Figure 3:**
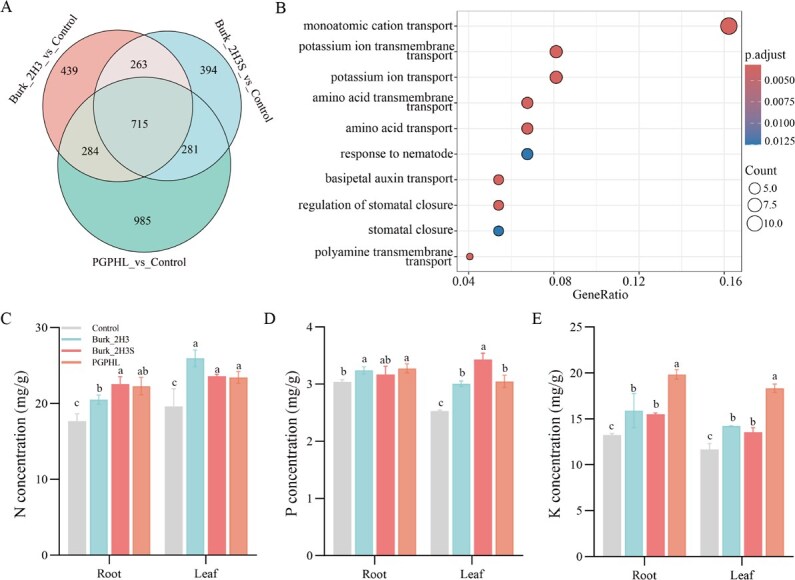
Transcriptome analysis of DEGs in citrus roots. (A) Venn diagram of DEGs in the comparison of control vs. Burk_2H3, control vs. Burk_2H3S and control vs. PGPHL. (B) GO analysis of up-regulation DEGs shared by citrus roots inoculated with Burk_2H3, Burk_2H3S and PGPHL. Mineral elements of nitrogen (C), phosphorus (D) and potassium (E) in roots and leaves of citrus plants inoculated with Burk_2H3, Burk_2H3S, or PGPHL strains were quantified. Data are expressed as mean ± standard deviation of 4 independent biological replicates. (Tukey's HSD test).

Transcriptomic analysis of citrus roots treated with Burk_2H3, Burk_2H3S, or PGPHL identified 715 shared differentially expressed genes (DEGs) (Fig. 3A, Table S5). Among these, 63 were transmembrane transport-related genes, with 41 upregulated and 22 downregulated (Fig. S6B). To validate these findings, we performed RT-qPCR on 11 representative transporter genes. The results were consistent with the RNA-seq data: eight genes, including nitrate transporter *FhNRT2.1*, amino acid transporter *FhAVT1C*, phosphate transporters *FhPHO1.1* and *FhPHO1.2*, potassium channel *FhKAT1*, potassium transporters *FhHAK5* and *FhKT2*, and NRT1/PTR family protein *FhPTR2*, were significantly upregulated, while zinc transporter *FhZIP4*, ammonium transporter *FhAMT2*, and copper transporter *FhCOPT1* were downregulated (Fig. S7A–K). GO enrichment further confirmed the upregulation of monoatomic cation transporters (Fig. 3B).

Consistent with these transcriptional changes, all three treatments enhanced nutrient acquisition. Leaves showed elevated levels of nitrogen (N), potassium (K), phosphorus (P), calcium (Ca), and manganese (Mn), while roots accumulated higher N, P, and K (Fig. 3C–E, Fig. S8A and E). In addition, Burk_2H3S and PGPHL increased root sulfur (S) content, and PGPHL specifically reduced sodium (Na^+^) levels (Fig. S8B and H). Together, these results demonstrate that Burk_2H3, Burk_2H3S, and PGPHL activate the transcription of transmembrane transport-related genes and promote nutrient uptake in citrus roots.

### Field fertilization of PGPHL significantly increases plant biomass and fruit yield

**Figure 4:**
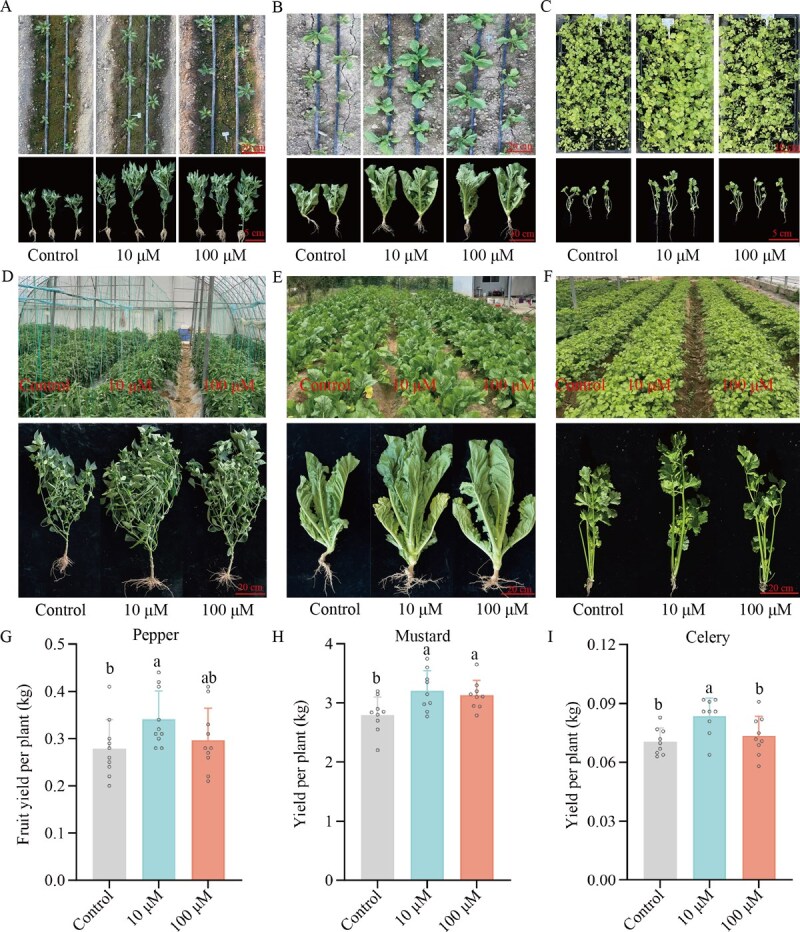
Field treatment of pepper, mustard and celery with PGPHL. The growth performance of pepper, mustard and celery plants were recorded at two weeks after PGPHL treatment (A–C) and at maturity (D–F). Yield parameters (G–I) of pepper, mustard, and celery plants upon PGPHL inoculation at maturity were recorded. Data are expressed as mean ± standard deviation of 10 independent biological replicates (Tukey's HSD test).

Despite their potential to boost crop productivity, PGPR face significant field application challenges, such as low viability, storage and transport instability, and inconsistent performance in varied agricultural settings [22]. Therefore, microbial-derived bioactive metabolites, which are inherently stable and have broad adaptability, represent a highly promising alternative for overcoming these limitations. To enable agricultural application of the bioactive metabolite PGPHL, we synthesized PGPHL via a hexanoic acid-derived pathway (Fig. S9). ^1^H NMR and HPLC confirmed structural identity and high purity (99.68%, Fig. S10A and B). Field trials at 10 μM PGPHL significantly increased pepper yield (20.79%), mustard biomass (14.54%), and celery biomass (18.31%) whereas 100 μM PGPHL significantly increased mustard yield (11.90%) but showed no efficacy for pepper and celery (Fig. 4), indicating dose-dependent activity.

## Discussion

Our previous screening identified Burk_2H3 as a PGPR candidate with growth-promoting potential under tissue culture conditions [21]. Here we show it promotes growth across multiple plant species, and metabolomic and functional assays identify PGPHL, an N-acyl-homoserine lactone (AHL), as the key metabolite mediating this effect, shedding light on the mechanism of PGPR–plant interaction.

AHLs, the most well-characterized QS signals, consist of a conserved homoserine lactone ring and a variable acyl chain [23, 24]. Unlike canonical PGPR secretions, such as Indole-3-acetic acid (IAA), siderophores, or 1-aminocyclopropane-1-carboxylic acid (ACC) deaminase, which directly promote plant growth [5, 6], AHLs function as interkingdom signals rather than as direct nutrients or hormones. They exert growth-promoting effects indirectly by modulating host metabolism, including enhancing photosynthetic pigment levels, rebalancing endogenous hormones, and inducing defense responses [25, 26]. Their functional specificity arises from structural variations in the acyl moiety, particularly the chain length (C4–C14) and substitutions at the 3-carbon position [27]. Short-chain AHLs (C4–C8) are predominantly associated with plant growth promotion [28, 29], whereas long-chain variants (C10–C14) often prime stress adaptation [30, 31].

As a short-chain AHL, PGPHL (3-Oxo-C8-HSL) has been reported to induce root growth primarily via the G protein-coupled receptors (GPCR) signaling pathway in *Arabidopsis thaliana* [32]. However, it looks the PGPHL employed a different strategy to promote plant growth in citrus, since no response of GPCR to PGPHL was observed in our transcriptome data (Table S5). Instead, we discovered that PGPHL significantly upregulates a suite of genes encoding transporters for a broad spectrum of mineral nutrients, including *FhNRT2.1*, *FhPHO1.1*, *FhKT2*, as well as proteins involved in calcium and sulfur transport (Figs S6B and S7). The concerted upregulation of these transporters, corroborated by the elevated tissue levels of nitrogen, phosphorus, and potassium (Fig. 3B–E), that PGPHL-mediated plant growth promotion may be associated with enhanced root nutrient uptake capacity.

While PGPR hold promise for enhancing crop productivity, their field efficacy is often constrained by challenges, such as poor bacterial viability, storage and transportation instability, and inconsistent performance across diverse crops and soil types [33, 34]. To overcome these limitations, microbial-derived bioactive compounds with enhanced stability and broad adaptability represent a promising alternative. In this study, we identified PGPHL and demonstrated its broad-spectrum growth-promoting function. The PGPHL not only stimulated growth in citrus and tomato under pot conditions (Fig. 2B–D, Fig. S4) but also significantly increased the biomass (or yield) of multiple horticultural crops in field trials (Fig. 4). Future studies will focus on elucidating the molecular mechanisms underlying PGPHL's crop-specific growth-promoting effects, and optimizing its synthesis to develop cost-effective production for large-scale agricultural applications.

In conclusion, this study characterized the citrus rhizosphere *Burkholderia* sp. Burk_2H3 and identified PGPHL as its key bioactive metabolite. Mechanistically, our findings suggest that PGPHL-promoted plant growth may be associated with its facilitation of root acquisition of N, P, and K. Importantly, the efficacy of PGPHL extends beyond citrus, as field trials confirmed its capacity to significantly enhance the biomass and yield of diverse horticultural crops. Our findings provide new insights into rhizobacterial modulation of plant performance and will help develop next-generation microbial agrochemicals for sustainable agriculture.

## Materials and methods

### Isolation of bacterial strains from citrus rhizosphere soils

Bacterial strains were isolated according to established protocols [21]. Citrus rhizosphere soil samples were suspended in sterile PBS buffer and subjected to serial dilution at three concentration gradients (500×, 1000×, and 10 000×). Single strains were isolated using the colony-picking method on agar plates: 20 to 25 ml of agar medium was poured into Petri dishes, and diluted samples were spread onto the surface, followed by incubation at 30°C for 72 h. After single colonies developed, preliminary screening was performed based on morphological characteristics. Further identification via 16S rRNA gene sequencing confirmed the isolation of the single *Burkholderia* strain Burk_2H3, which was subsequently used for plant co-cultivation studies.

### Bacterial inoculation in pot-culture and measurement of plant growth

Seedlings of citrus (*Fortunella hindsii*), tomato (*Solanum lycopersicum*, Micro-tom), and thale cress (*Arabidopsis thaliana*) were cultured in pots with compound substrates (peat soil: vermiculite = 1:1) in a greenhouse. Citrus seeds were surface-sterilized with 2% NaOCl and then sown in autoclaved vermiculite. They were grown in a growth chamber at 28°C under a 16-h light/8-h dark photoperiod for one month to obtain seedlings. Tomato and thale cress seeds were surface-sterilized with 2% NaOCl for 15 min, transferred to agar plates, covered with foil, and vernalized at 4°C for 2 days with the plate upside down. The plates were then incubated in an incubator at 28°C with a photoperiod of 16-h light /8-h dark for 7 days to obtain seedlings.

Prior to inoculation, citrus seedlings were cultivated in a greenhouse, while tomato and thale cress seedlings were grown in a growth chamber at 22°C under a 16-h light/8-h dark photoperiod. Bacterial strains were first grown on LB agar plates for 3 days. Single colonies were then inoculated into liquid LB medium and cultured for 12 h. For rhizosphere inoculation, either 5 ml of bacterial suspension (OD_600_ = 0.2) or 5 ml of cell-free supernatant (obtained by filtering the bacterial culture through a 0.2-μm membrane) was applied to the rhizosphere of each plant. Inoculations were performed every 7 days for a total of three applications. Plant height was measured at 4 weeks post-inoculation for tomato and thale cress, and at 8 weeks post-inoculation for citrus.

For PGPHL treatment, the citrus seedlings were pot-cultured in a greenhouse, the tomato and thale cress seedlings were pot-cultured in a growth chamber at 22°C with a photoperiod of 16-h light /8-h dark. PGPHL and two control compounds, Ametryn and Vinclozolin, were purchased from Macklin Biochemical Co., Ltd. (Shanghai, China). The other two control substances, the oligopeptides Val–Leu–Thr–Gly–Ser (VLTGS) and Thr–Val–Lys–Glu (TVKE), were synthesized by GenScript Biotech Corporation (Nanjing, China). All five compounds were dissolved in distilled water to prepare 10 μM stock solutions. A 5-ml aliquot of each solution was inoculated into the rhizosphere of 1-month-old potted citrus plants, 2-week-old tomato plants, and 2-week-old thale cress plants, respectively. The inoculation was performed every 7 days for a total of three times. The heights of tomato and thale cress plants were measured at 4 weeks post–pot culture, and citrus plants at 8 weeks.

### Untargeted metabolomic analysis of bacterial secreta

Untargeted metabolomic analysis was performed on the bacterial secretome of strain Burk_2H3, with the secretomes of three non–plant-growth-promoting (non-PGPR) strains (Burk_1F1, Burk_2A1, and Burk_2H2) [21] serving as negative controls, as previously described [35]. Briefly, 6 ml of cell-free supernatants was freeze-dried by vacuum and ground to a powder, and then the Burk_2H3S powder was suspended in 1.2 ml methanol (70%). After homogenization, the suspension was sonicated in an ice bath for 10 min, followed by centrifugation (10 000 × g) at 4°C for 30 min. The resulting supernatant was filtered using a microporous membrane (0.22 μm pore size) for UPLC–MS/MS detection (Thermo Scientific, USA). The resulting solution was separated in mobile phase A (water containing 0.1% formic acid) and mobile phase B (acetonitrile containing 0.1% formic acid) by a Waters ACQUITY UPLC HSS T3 Column (2.1 mm × 100 mm, 1.8 μm). The flow rate of the mobile phases was set as 0.40 ml/min. The raw UPLC–MS/MS data were subjected to processing of peak integration, calibration, and metabolite quantification using TMBQ software (v1.0, Metabo-Profile, Shanghai, China). The metabolites were clustered by multivariate statistical analysis (Partial Least Squares-Discriminant Analysis, PLS-DA), and differential metabolites between the three negative controls (Burk_1F1S, Burk_2A1S, and Burk_2H2S) and the Burk_2H3S group were identified using univariate statistical analyses (Student's *t*-test and Mann–Whitney–Wilcoxon *U* test).

### Transcriptomic analysis of strain/secreta-inoculated citrus roots

At 8 weeks post pot culture, citrus roots treated with or without the bacterial strain Burk_2H3, Burk_2H3 secreta (Burk_2H3S), PGPHL and LB liquid medium (control) were collected from pot cultured plants, and then snap-frozen in liquid nitrogen for RNA extraction and sequencing. Each pot was planted with three citrus seedlings. The seedlings within each pot were pooled to form one biological replicate sample. Four such biological replicates were established per treatment group.

Total RNA of the citrus roots was extracted using the FastPure® Plant Total RNA Isolation Kit (Vazyme Biotech Co., Ltd, Nanjing, China), and RNA-seq libraries were constructed by Novogene (Beijing, China). Sequence alignment was carried out with STAR software against the *Fortunella hindsii* v1.0 reference genome (http://citrus.hzau.edu.cn/).

Gene expression levels were calculated as fragments per kilobase of transcript per million mapped reads (FPKM) using HTSeq [36]. Heatmaps of DEGs were plotted, and their relative expression levels were quantified using pheatmap, dplyr, and ggplot packages in R (version 4.3.0). Based on the resulting heatmaps, cluster analysis was performed, and optimal cluster number was determined using the total within-cluster sum of square. The DEGs between the Burk_2H3, Burk_2H3S, and PGPHL treatment groups versus the Control group were set as an adjusted *P* < 0.05 and |log2 FC (fold change)|>1, and Gene Ontology (GO) analysis was performed using the R package topGO (version: 4.0).

### Determination of mineral nutrients in citrus roots and leaves

The roots and leaves of citrus seedlings inoculated with/without Burk_2H3, Burk_2H3S and PGPHL were digested, according to a previous method [37].

H₂SO₄–H₂O₂ digestion method (for N/P determination): Weigh 0.10–0.15 g dried citrus root or leaf sample into a 50 mL digestion tube. Moisten with two to three drops water, add 5 ml conc. H₂SO₄, swirl to wet, and stand overnight. Preheat IR furnace to 160°C (15 min), place tube in furnace; heat until white fumes evolve (swirl frequently). When solution turns uniform brown-black, remove and cool slightly. Slowly add 6 drops 30% H₂O₂ (swirl gently until reaction subsides), return to furnace; heat to gentle boil and digest 15 min. Remove, cool slightly. Repeat H₂O₂ addition (decreasing volume each time and digesting at gentle boil for 15 min), cooling two to three times until solution is near-colorless. Digest at gentle boil 10 min more to decompose residual H₂O₂. Cool to room temperature. Carefully add water to 1/2–2/3 volume; mix, dilute to 50 ml mark. Filter through dry qualitative filter paper; use filtrate [37].

HNO₃–HClO₄ digestion method (4:1, V/V): Weigh 0.1 to 0.15 g of dried citrus root/leaf sample into a 50 ml digestion tube. Add 10 ml of concentrated HNO₃–HClO₄ mixed acid (4:1, V /V) and shake gently. Place the tube in an infrared digestion furnace and digest at 160°C until yellow-brown fumes dissipate and solids dissolve completely. Then increase temperature to 240°C for further digestion; during this stage, carefully rotate the tube two to three times to wash down solids adhering to the inner walls. Continue digestion until the solution becomes colorless and transparent. Remove the tube, cool to room temperature, dilute to 50 ml with deionized water, and mix thoroughly. Filter through quantitative filter paper; the filtrate is ready for use [37].

The P concentration was determined using a molybdenum antimony colorimetric method [38]. The N concentration was determined using a Kjeldahl method [39]. The concentrations of mineral nutrients K, B, Ca, Cu, Fe, Mg, Mn, S, and Zn were determined using an Agilent 5100 SVDV ICP-OES (Agilent Technologies) [40].

### Synthesis of PGPHL

PGPHL was synthesized according to a literature procedure [41], with the following steps:

Step 1: A solution of hexanoic acid (178 g, 1.52 mol) in THF (2.5 L) was treated with CDI (296 g, 1.83 mol) and stirred at 25°C for 1 h. Subsequently, potassium 3-methoxy-3-oxopropanoate (355 g, 2.89 mol) and MgCl₂ (124.3 g, 1.83 mol) were added slowly, and stirring was continued for 4 h at 25°C. After filtration and concentration, the residue was partitioned between water (2.0 L) and ethyl acetate. The organic layer was washed sequentially with 1 M HCl and saturated Na₂CO₃ solution, dried over Na₂SO₄, and concentrated to afford crude compound 3 (Fig. S9).

Step 2: A mixture of 3 (100 g, 0.58 mol) and ethylene glycol (216 g, 3.48 mol) in DCM (1 L) was treated dropwise with TMSCl (252 g, 2.32 mol) and stirred at 25°C for 1 h. The reaction was quenched with water (500 mL), extracted with DCM, dried (Na₂SO₄), and concentrated to give compound 4 (Fig. S9).

Step 3: To a solution of 4 (120 g, 0.56 mol) in MeOH (1.2 L) was added a solution of LiOH·H₂O (252 g, 2.32 mol) in water (600 mL). The mixture was stirred at 25°C for 4 h. After removal of methanol, the aqueous residue was acidified to pH 4–5 with 4 N HCl. The product was extracted into DCM, dried (Na₂SO₄), and concentrated to yield crude compound 5 (Fig. S9).

Step 4: A mixture of 5 (156 g, 0.77 mol) and EDCI (163.1 g, 0.85 mol) in anhydrous DCM (1.5 L) was stirred under N₂ for 30 min. Compound 6 (128 g, 0.93 mol) and DMAP (161 g, 1.32 mol) were then added. After stirring overnight, the mixture was diluted with DCM (1.0 L), washed with 1 N HCl, dried (Na₂SO₄), and concentrated. Purification by column chromatography afforded compound 7 (Fig. S9).

Step 5: Compound 7 (65 g, 0.23 mol) was dissolved in a mixture of TFA and water (460 mL/230 mL) and stirred under N₂ until the reaction was complete (monitored by TLC). The mixture was carefully quenched with saturated NaHCO₃ solution, extracted with DCM, dried (Na₂SO₄), and concentrated. Final purification by column chromatography yielded PGPHL. Its structure was confirmed by ^1^H NMR and ESI–HRMS analysis (Fig. S10).

### Field validation of PGPHL efficacy

A field experiment was conducted at the Experimental Field of the Vegetable and Flower Research Institute in Ganzhou City, Jiangxi Province, China (25°49′2.039′′N, 114°40′11.339′′E) to investigate the effects of PGPHL (10 and 100 μM) on the yield of pepper (*Capsicum annuum*), celery (*Apium graveolens*), and mustard (*Brassica juncea*). A water-treated group served as the control (Control), with 2000 plants per crop assigned to each concentration treatment. On the day of transplantation, each plant was irrigated with 200 ml of either water or 200 ml PGPHL solution, followed by additional irrigations every 15 days for a total of three applications. After maturation the yield was calculated by weighing the harvested produce: fruit weight was measured for pepper, while whole plant fresh weight was recorded for leafy vegetables (celery and mustard).

### Statistical analysis

Statistical analyses were conducted using GraphPad Prism 9 (GraphPad Software Inc., La Jolla, CA, USA) and SPSS 29.0 (SPSS, Chicago, IL, USA). Data are presented as the mean ± standard deviation, with no fewer than six biological replicates per experiment. Significant differences between groups were assessed using Student's *t*-test for pairwise comparisons and Tukey's HSD test for all pairwise comparisons among multiple groups. A *P* value of less than 0.05 was considered statistically significant.

## Ethics approval and consent to participate

No ethics approval or consent to participate was required.

## Supplementary Material

Web_Material_uhag071

## Data Availability

The RNA-seq datasets deposited in SRA database under BioProject number PRJNA 1272145 (https://www.ncbi.nlm.nih.gov/bioproject/PRJNA1272145).

## References

[1] Chieb M, Gachomo EW. The role of plant growth promoting rhizobacteria in plant drought stress responses. BMC Plant Biol. 2023;23:407 37626328 10.1186/s12870-023-04403-8 PMC10464363

[2] Egamberdieva D, Kamilova F, Validov S. et al. High incidence of plant growth-stimulating bacteria associated with the rhizosphere of wheat grown on salinated soil in Uzbekistan. Environ Microbiol. 2008;10:1–9 18211262 10.1111/j.1462-2920.2007.01424.x

[3] Fan Z, Wu Y, Zhao L. et al. MYB308-mediated transcriptional activation of plasma membrane H+-ATPase 6 promotes iron uptake in citrus. Hortic Res. 2022;9:uhac088 35685222 10.1093/hr/uhac088 PMC9171118

[4] Haque M, Islam S, Sheikh MA. et al. Quorum sensing: a new prospect for the management of antimicrobial-resistant infectious diseases. Expert Rev Anti-Infect Ther. 2021;19:571–86 33131352 10.1080/14787210.2021.1843427

[5] Sun X, Xu Z, Xie J. et al. *Bacillus velezensis* stimulates resident rhizosphere *Pseudomonas stutzeri* for plant health through metabolic interactions. ISME J. 2022;16:774–87 34593997 10.1038/s41396-021-01125-3 PMC8483172

[6] Wang N, Wang T, Chen Y. et al. Microbiome convergence enables siderophore-secreting-rhizobacteria to improve iron nutrition and yield of peanut intercropped with maize. Nat Commun. 2024;15:839 38287073 10.1038/s41467-024-45207-0 PMC10825131

[7] Backer R, Rokem JS, Ilangumaran G. et al. Plant growth-promoting rhizobacteria: context, mechanisms of action, and roadmap to commercialization of bio stimulants for sustainable agriculture. Front Plant Sci. 2018;9:1473–89 30405652 10.3389/fpls.2018.01473 PMC6206271

[8] Harbort CJ, Hashimoto M, Inoue H. et al. Root-secreted coumarins and the microbiota interact to improve iron nutrition in *Arabidopsis*. Cell Host Microbe. 2020;28:825–837.e6 33027611 10.1016/j.chom.2020.09.006 PMC7738756

[9] Zhang L, Feng G, Declerck S. Signal beyond nutrient, fructose, exuded by an arbuscular mycorrhizal fungus triggers phytate mineralization by a phosphate solubilizing bacterium. ISME J. 2018;12:2339–51 29899507 10.1038/s41396-018-0171-4 PMC6155042

[10] Imran A, Mirza MS, Shah TM. et al. Differential response of kabuli and desi chickpea genotypes toward inoculation with PGPR in different soils. Front Microbiol. 2015;6:859 26379638 10.3389/fmicb.2015.00859 PMC4548240

[11] Hakim S, Naqqash T, Nawaz MS. et al. Asma imran rhizosphere engineering with plant growth-promoting microorganisms for agriculture and ecological sustainability. Front Sustain Food Syst. 2021;5:7157

[12] Hanif MK, Malik KA, Hameed S. et al. Growth stimulatory effect of AHL producing *Serratia* spp. from potato on homologous and non-homologous host plants. Microbiol Soc. 2020;238:126506 10.1016/j.micres.2020.126506 32540731

[13] Sharma SB, Sayyed RZ, Trivedi MH. et al. Phosphate solubilizing microbes: sustainable approach for managing phosphorus deficiency in agricultural soils. Springer Plus. 2013;2:587 25674415 10.1186/2193-1801-2-587 PMC4320215

[14] Ghosh SK, Bera T, Chakrabarty AM. Microbial siderophore—a boon to agricultural sciences. Biol Control. 2020;144:104214

[15] Kumar V, Singh P, Jorquera MA. et al. Isolation of phytase-producing bacteria from Himalayan soils and their effect on growth and phosphorus uptake of Indian mustard (*Brassica juncea*). J Microbiol Biotechnol. 2013;29:1361–9 10.1007/s11274-013-1299-z 23546828

[16] Mukherjee A, Mazumder M, Verma A. et al. A bacterial signal coordinates plant-microbe fitness trade-off to enhance sulfur deficiency tolerance in plants. Cell Host Microbe. 2025;33:1748–1764.e6 41015044 10.1016/j.chom.2025.09.007

[17] Liu Y, Kyle S, Straight PD. Antibiotic stimulation of a *Bacillus subtilis* migratory response. MSphere. 2018;3:e0058617 10.1128/mSphere.00586-17 PMC5821984 29507890

[18] Mun BG, Lee WH, Kang SM. et al. LH 4 promotes plant growth and resistance against *Sclerotinia sclerotiorum* in cucumber via modulation of enzymatic and defense pathways. Plant Soil. 2020;448:87–103

[19] Wang N, Pierson EA, Setubal JC. et al. The Candidatus Liberibacter–host interface: insights into pathogenesis mechanisms and disease control. Annu Rev Phytopathol. 2017;55:451–82 28637377 10.1146/annurev-phyto-080516-035513

[20] Darriaut R, Lailheugue V, Masneuf-Pomarède I. et al. Grapevine rootstock and soil microbiome interactions: keys for a resilient viticulture. Hortic Res. 2022;9:uhac019 35184168 10.1093/hr/uhac019 PMC8985100

[21] Li H, Wang Y, Deng L. et al. Microbial biobank-based strain phenotyping efficiently identifies plant growth-promoting bacteria from citrus rhizosphere. Hortic Adv. 2025;3:3

[22] Russ D, Fitzpatrick CR, Teixeira P. et al. Deep discovery informs difficult deployment in plant microbiome science. Cell. 2023;186:4496–513 37832524 10.1016/j.cell.2023.08.035

[23] Schenk ST, Hernández-Reyes C, Samans B. et al. N-acyl-homoserine lactone primes plants for cell wall reinforcement and induces resistance to bacterial pathogens via the salicylic acid/oxylipin pathway. Plant Cell. 2014;26:2708–23 24963057 10.1105/tpc.114.126763 PMC4114961

[24] Jamil F, Mukhtar H, Fouillaud M. et al. Rhizosphere signaling: insights into plant-rhizomicrobiome interactions for sustainable agronomy. Microorganisms. 2022;10:899 35630345 10.3390/microorganisms10050899 PMC9147336

[25] Mathesius U, Mulders S, Gao M. et al. Extensive and specific responses of a eukaryote to bacterial quorum-sensing signals. Proc Natl Acad Sci USA. 2003;100:1444–9 12511600 10.1073/pnas.262672599 PMC298792

[26] Babenko LM, Kosakivska IV, Romanenko KO. Molecular mechanisms of N-acyl homoserine lactone signals perception by plants. Cell Biol Int. 2022;46:523–34 34937124 10.1002/cbin.11749

[27] Middleton B, Rodgers HC, Cámara M. et al. Direct detection of N-acylhomoserine lactones in cystic fibrosis sputum. FEMS Microbiol Lett. 2002;207:1–7 11886742 10.1111/j.1574-6968.2002.tb11019.x

[28] von Rad U, Klein I, Dobrev PI. et al. Response of *Arabidopsis thaliana* to N-hexanoyl-dl-homoserine-lactone, a bacterial quorum sensing molecule produced in the rhizosphere. Planta. 2008;229:73–85 18766372 10.1007/s00425-008-0811-4

[29] Pazarlar S, Cetinkaya N, Bor M. et al. N-acyl homoserine lactone-mediated modulation of plant growth and defense against *Pseudoperonospora cubensis* in cucumber. J Exp Bot. 2020;71:6638–54 32822478 10.1093/jxb/eraa384

[30] Götz-Rösch C, Sieper T, Fekete A. et al. Influence of bacterial N-acyl-homoserine lactones on growth parameters, pigments, antioxidative capacities and the xenobiotic phase II detoxification enzymes in barley and yam bean. Front Plant Sci. 2015;6:205 25914699 10.3389/fpls.2015.00205 PMC4392610

[31] Liu F, Bian Z, Jia Z. et al. The GCR1 and GPA1 participate in promotion of Arabidopsis primary root elongation induced by N-acyl-homoserine lactones, the bacterial quorum-sensing signals. Mol Plant-Microbe Interact. 2012;25:677–83 22250582 10.1094/MPMI-10-11-0274

[32] Custódio V, Salas-González I, Gopaulchan D. et al. Individual leaf microbiota tunes a genetic regulatory network to promote leaf growth. Cell Host Microbe. 2025;33:436–450.e15 40020663 10.1016/j.chom.2025.02.002

[33] Li J, Wang J, Liu H. et al. Application of microbial inoculants significantly enhances crop productivity: a meta-analysis of studies from 2010 to 2020. J Sustain Agric Environ. 2022;1:216–25

[34] Compant S, Cassan F, Kostić T. et al. Harnessing the plant microbiome for sustainable crop production. Nat Rev Microbiol. 2025;23:9–23 39147829 10.1038/s41579-024-01079-1

[35] Kruse S, Becker S, Pierre F. et al. Metabolic profiling of bacterial co-cultures reveals intermicrobiome interactions and dominant species. J Chromatogr A. 2023;1694:463911 36931138 10.1016/j.chroma.2023.463911

[36] Anders S, Pyl PT, Huber W. HTSeq—a python framework to work with high-throughput sequencing data. Bioinformatics. 2015;31:166–9 25260700 10.1093/bioinformatics/btu638 PMC4287950

[37] Hansen TH, Laursen KH, Persson DP. et al. Micro-scaled high-throughput digestion of plant tissue samples for multi-elemental analysis. Plant Methods. 2009;5:12 19781097 10.1186/1746-4811-5-12 PMC2761891

[38] Wu S, Zhang Y, Tian Q. et al. Biochar is superior to lime in improving acidic soil properties and fruit quality of *Satsuma mandarin*. Sci Total Environ. 2020;714:136722 31991273 10.1016/j.scitotenv.2020.136722

[39] Sáez-Plaza P, Navas MJ, Wybraniec S. et al. An overview of the kjeldahl method of nitrogen determination. Part II. Sample preparation, working scale, instrumental finish, and quality control. Anal Chem. 2013;43:224–72

[40] Marguí E, Dalipi R, Sangiorgi E. et al. Determination of essential elements (Mn, Fe, Cu and Zn) in herbal teas by TXRF, *FAAS and ICP-OES*. X-Ray Spectrom. 2022;51:204–13

[41] Hodgkinson JT, Galloway WRJD, Casoli M. et al. Robust routes for the synthesis of N-acylated-l-homoserine lactone (AHL) quorum sensing molecules with high levels of enantiomeric purity. Tetrahedron Lett. 2011;52:3291–4

