# Supplementary material for: Rhizobacteria promote plant growth via secretion of N-(3-oxooctanoyl)-L-homoserine lactone
Source: Hortic Res. 2026 Feb 28;13(6):uhag071. doi: 10.1093/hr/uhag071 (PMC13253340; doi:10.1093/hr/uhag071)
Supplement: Web_Material_uhag071 [file web_material_uhag071.zip › Supplementary Figure.pdf]

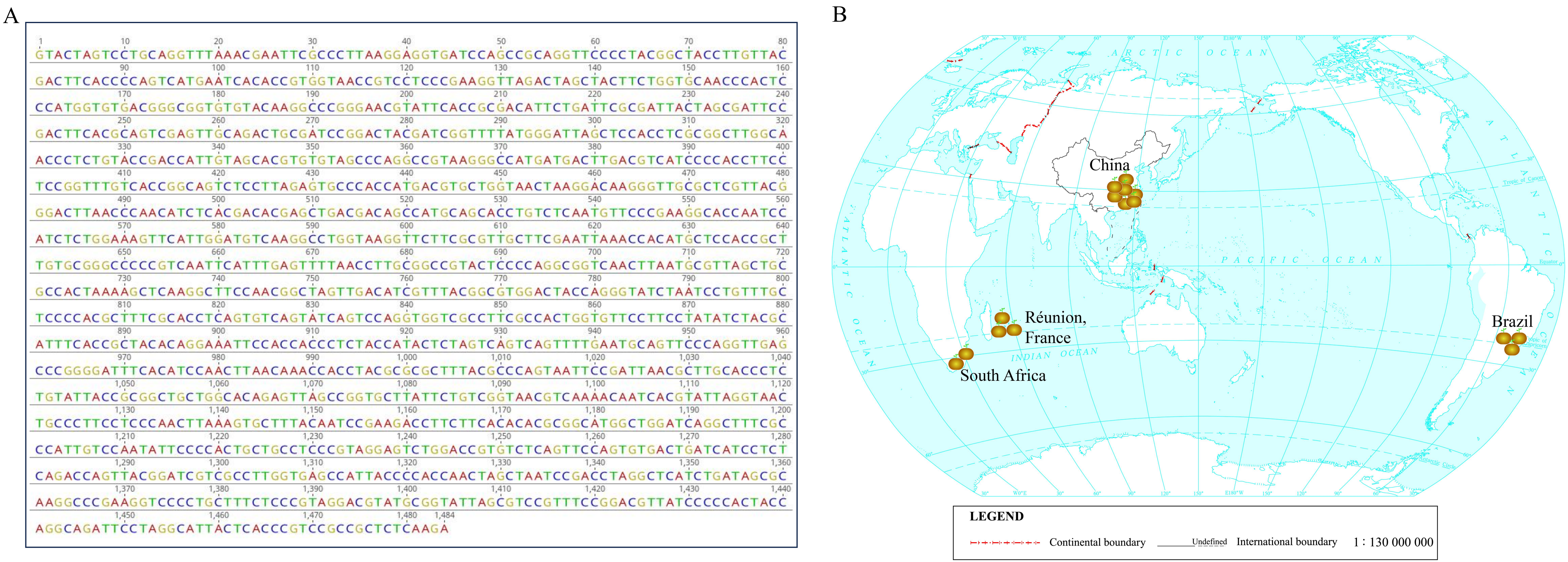

Fig. S1 16S rRNA sequence of strain Burk_2H3 and its distribution across diverse citrus habitats. (A) 16S rRNA sequence of strain Burk_2H3. The amplification primers were 27F: AGAGTTTGATCCTGGCTCAG and 1492R: TACGGYTACCTTGTTACGACTT. (B) Geographical distribution schematic diagram of Burk_2H3 across 15 citrus habitats worldwide. The 15 citrus habitats include 4 in China and 11 from a previous study of global citrus microbiome (Xu et al., 2018). The oranges in the picture represent citrus habitats. This figure is based on the standard map with the censor code: GS (2016) 2957.

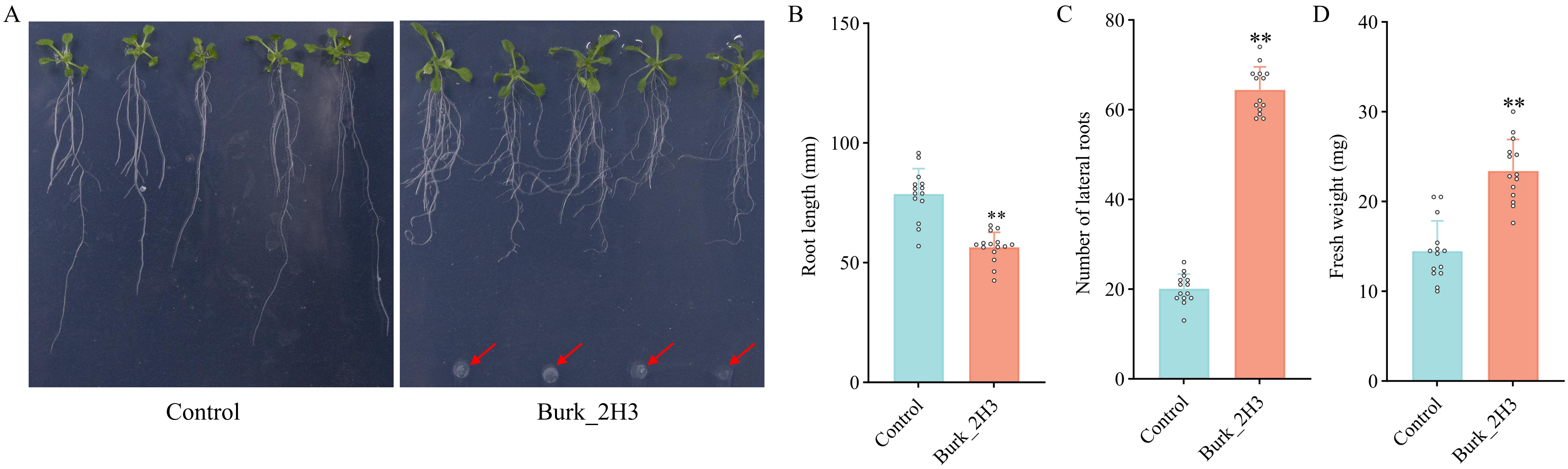

Fig. S2 Evaluation of the growth-promoting function of the strain 2H3. Effect of 2H3 inoculation on the growth of thale cress cultured in 1/2MS medium (A). The red arrows indicate the sites of bacterial inoculation. Statistical analysis was conducted on the root length (B), the number of lateral roots (C), and the fresh weight (D) of thale cress. Data are expressed as mean ± standard deviation of 15 independent biological replicates (15 thale cress plants). *, $p<0.05$; **, $p < 0.01$ (Student's *t*-test).

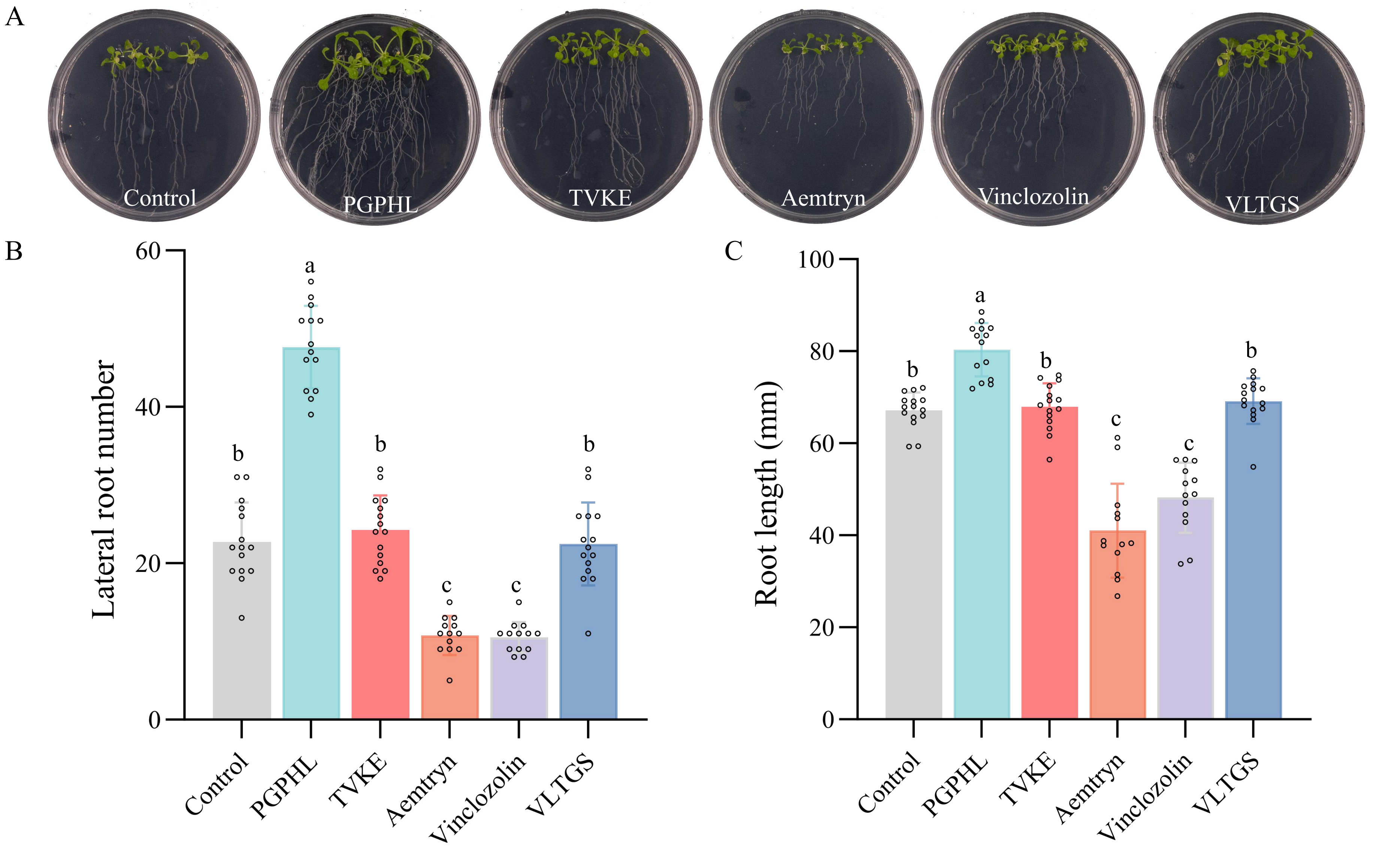

Fig. S3 Function verification of potential bioactive substances of Burk_2H3S in thale cress on 1/2MS medium. The growth performance of thale cress plants co-cultured with potential bioactive substances (A). Statistical analysis of lateral root number (B) and root length (C) of thale cress. PGHL: Plant Growth-Promoting Homoserine Lactone, VLTGS: Val-Leu-Thr-Gly-Ser, TVKE: Thr-Val-Lys-Glu. Data are expressed as mean ± standard deviation of 15 independent biological replicates (Tukey's HSD test).

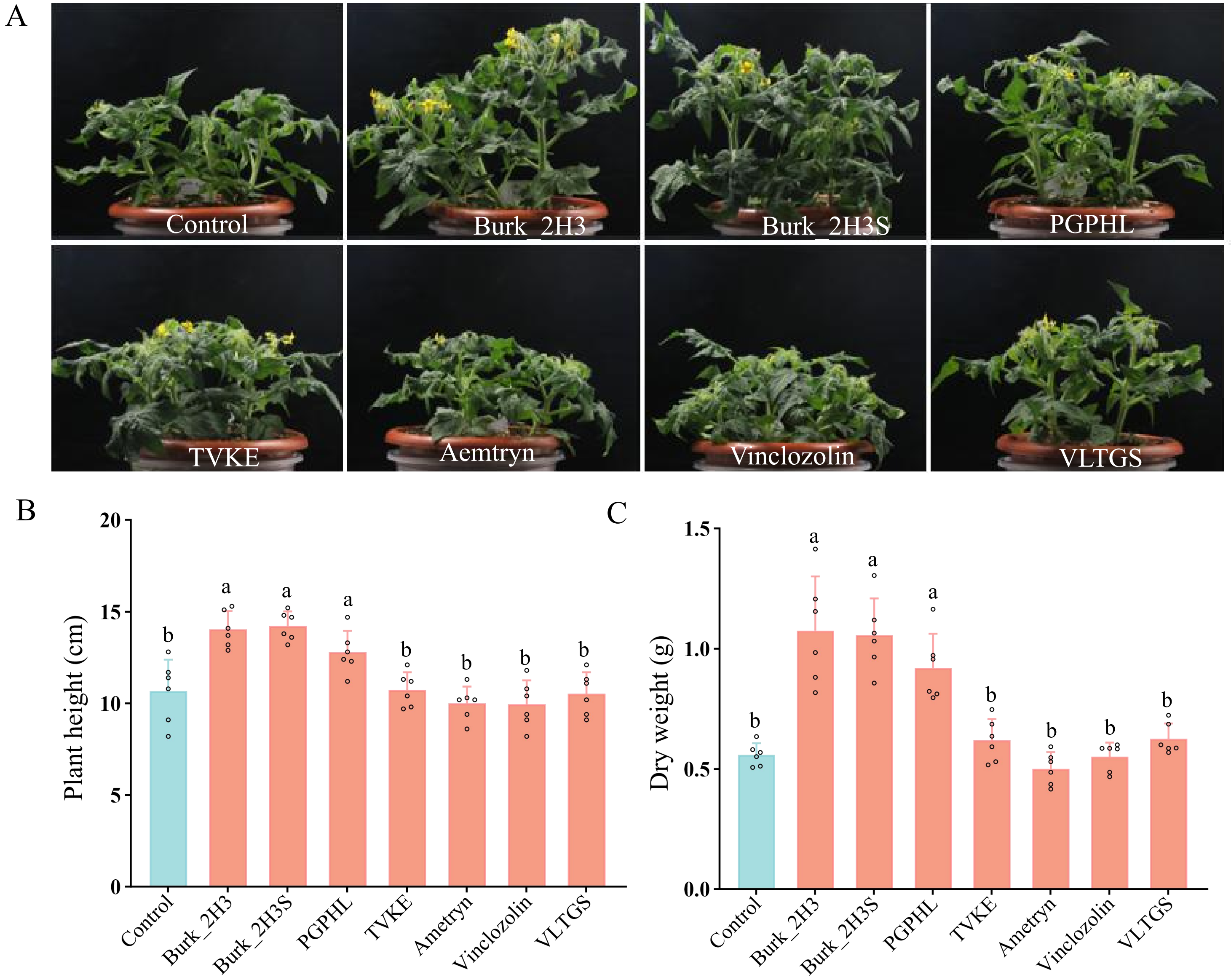

Fig. S4 Function verification of the potential bioactive substances of Burk_2H3S in pot cultured tomato. (A) The growth performance of tomato plants co-cultivated with potential bioactive substances. (B) Statistical analysis of plant height of tomato plants. (C) Statistical analysis of dry weight of tomato plants. Data are expressed as mean ± standard deviation of 12 independent biological replicates (Tukey's HSD test).

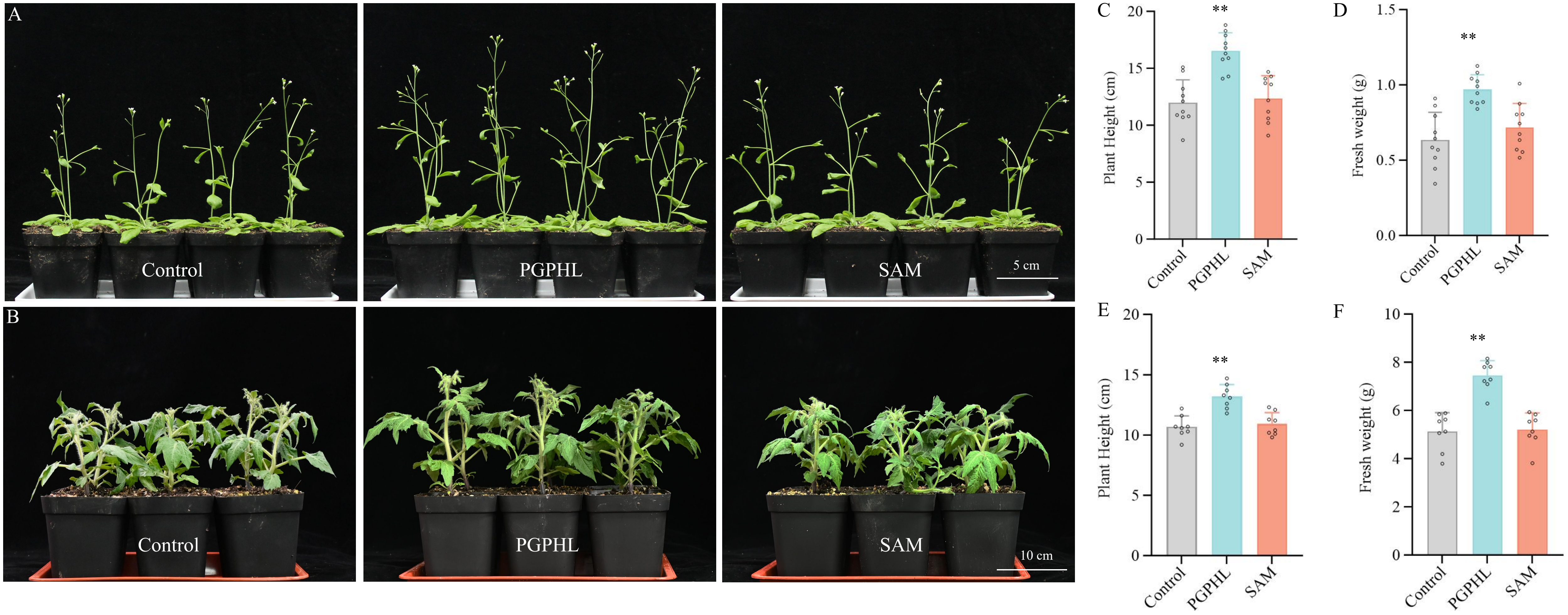

Fig. S5 Growth status of potted thale cress (A) and tomato (B) plants inoculated with PGPHL or SAM. Plant height (C, E) and fresh weight (D, F) were determined at 5 weeks post-inoculation for tomato and 4 weeks post-cultivation for thale cress. Data are expressed as mean ± standard deviation of 10 or 8 independent biological replicates (10 Arabidopsis or 8 tomato plants). *, $p<0.05$; **, $p < 0.01$ (Student's *t*-test).

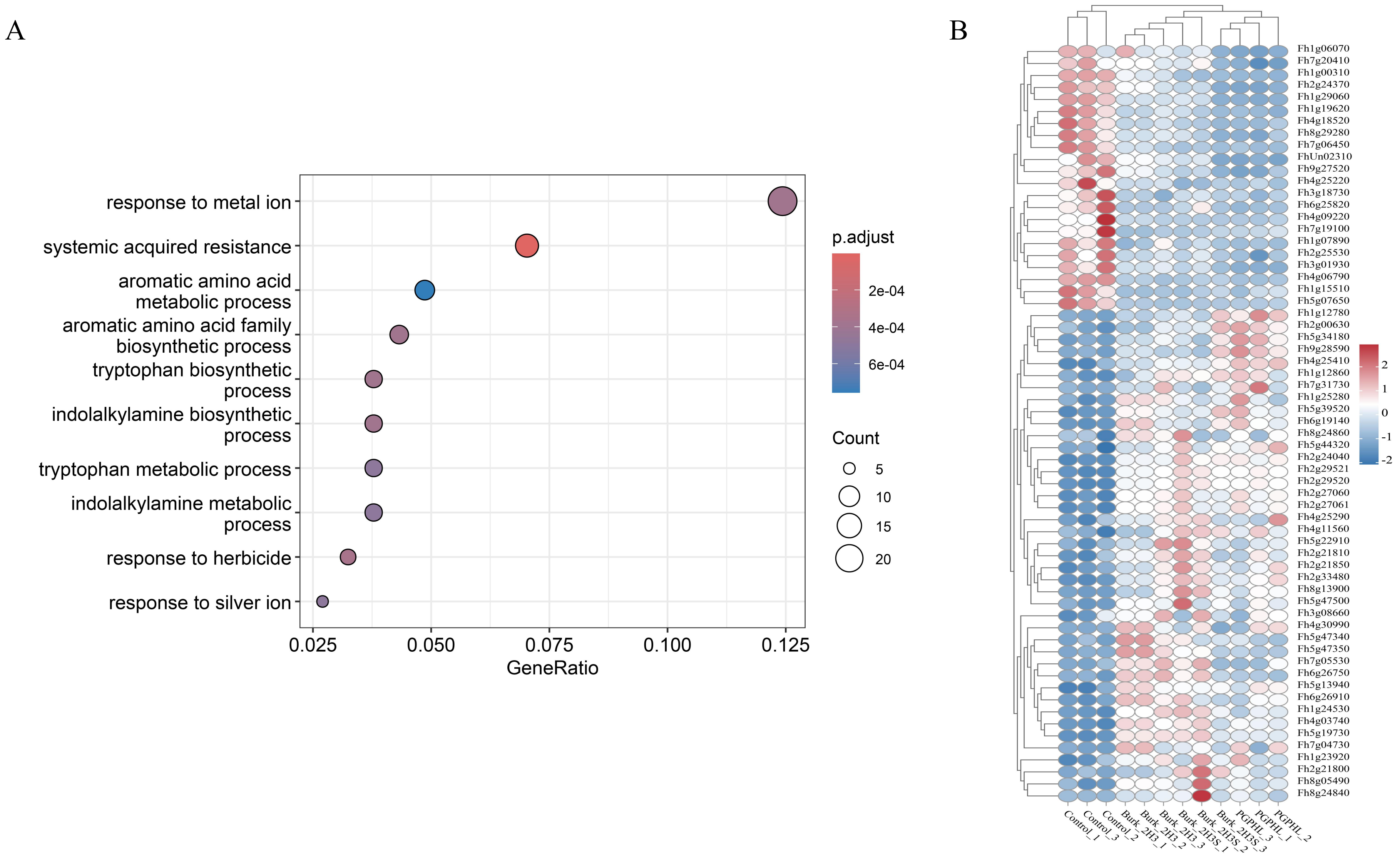

Fig. S6 Transcriptome analysis of differentially expressed genes (DEGs) in citrus roots. (A) GO analysis of down-regulation DEGs shared by citrus roots inoculated with Burk_2H3, Burk_2H3S and PGPHL. (B) Heatmap of the DEGs related to various mineral nutrients uptake and transport in citrus roots shared by two comparisons of control vs. Burk_2H3, control vs. Burk_2H3S and control vs. PGPHL. The transition from red to blue represents the gradual decrease in gene expression.

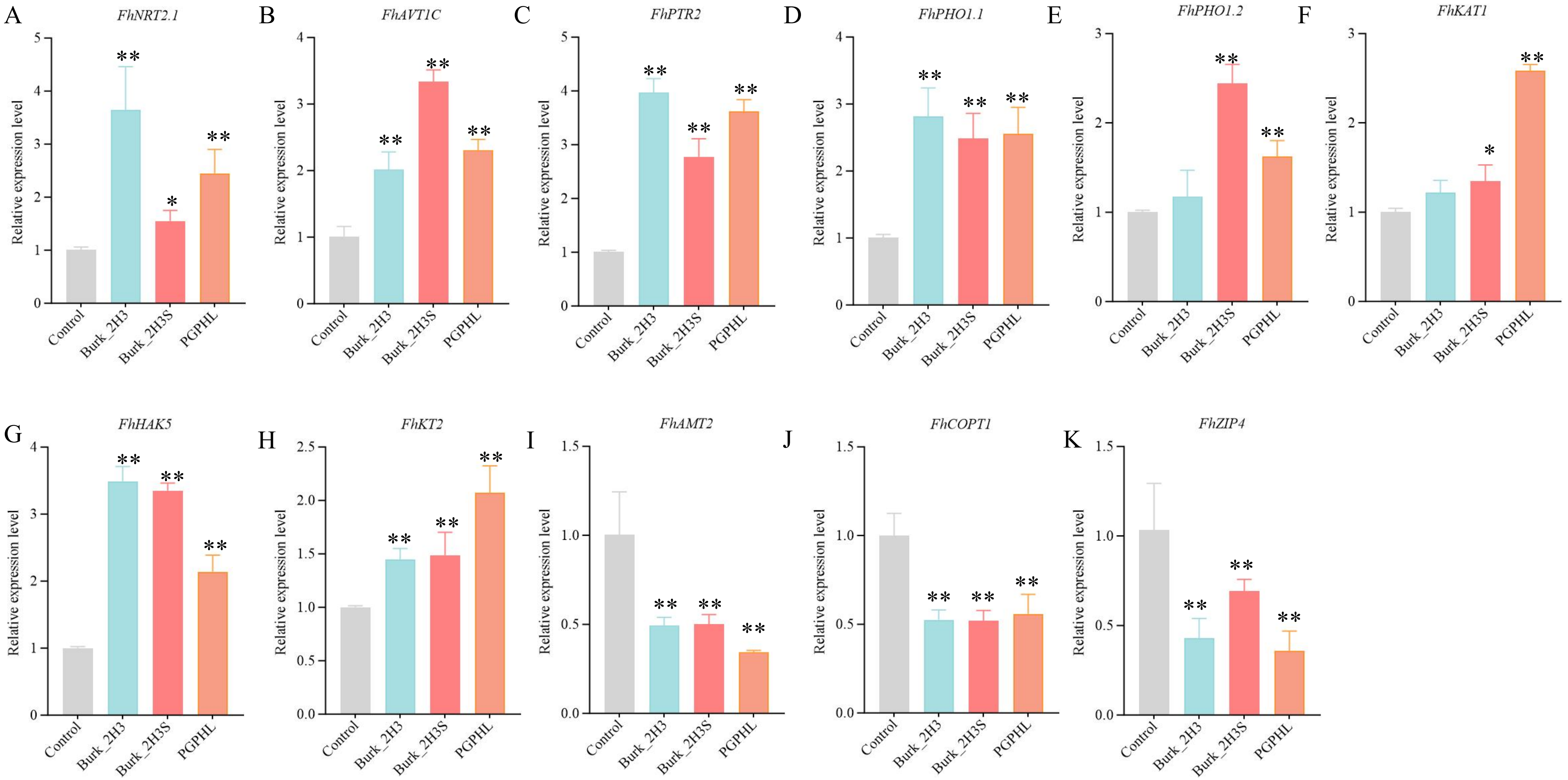

Fig. S7 Expression of nutrient transporter genes in treated citrus roots. RT-qPCR analysis shows relative expression of nitrate transporter *FhNRT2.1* (A), amino acid transporter *FhAVT1C* (B), NRT1/PTR family protein *FhPTR2* (C), phosphate transporters *FhPHO1.1* (D) and *FhPHO1.2* (E), potassium channel *FhKAT1* (F), potassium transporters *FhHAK5* (G) and *FhKT2* (H), zinc transporter *FhZIP4* (I), ammonium transporter *FhAMT2* (J), and copper transporter *FhCOPT1* (K) in roots treated with Burk_2H3, Burk_2H3S, or PGPHL. Expression was normalized to *Cs1g05000.1* (ΔCt method). Relative copy number (RCN) = $2^{-\Delta Ct} \times 100$, where ΔCt = Ct(target) – Ct(reference). Fold change was calculated by $2^{-\Delta\Delta Ct}$. Data are mean ± SD (n=3). *p<0.05, **p<0.01 (Student's *t*-test).

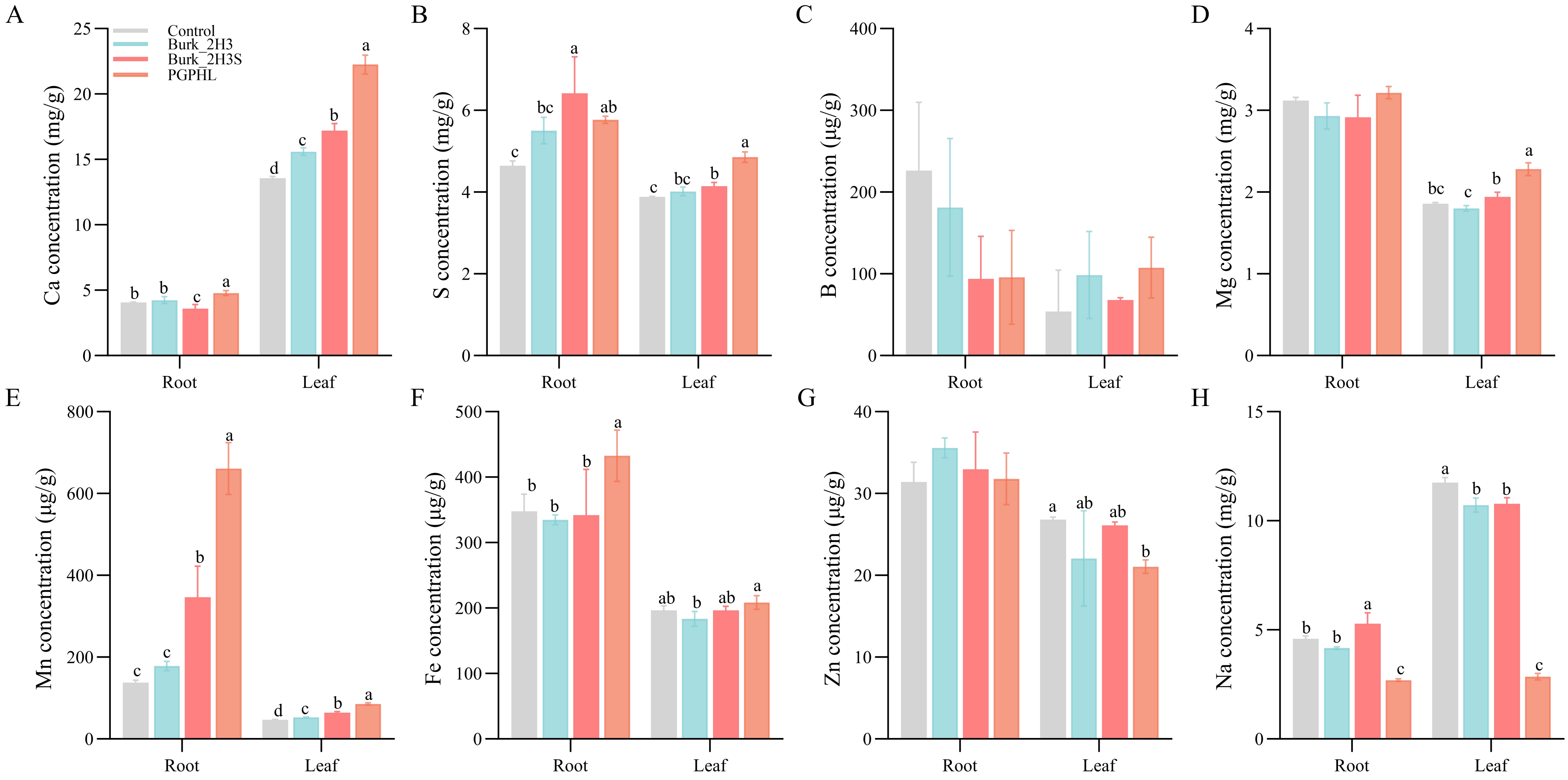

Fig. S8 Mineral element concentrations in roots and leaves of citrus plants inoculated with Burk_2H3, Burk_2H3S or PGPHL. The examined elements include calcium (A), sulfur (B), boron (C), magnesium (D), manganese (E), iron (F), zinc (G) and sodium (H). Data are expressed as mean ± standard deviation of 4 independent biological replicates. (Tukey's HSD test).

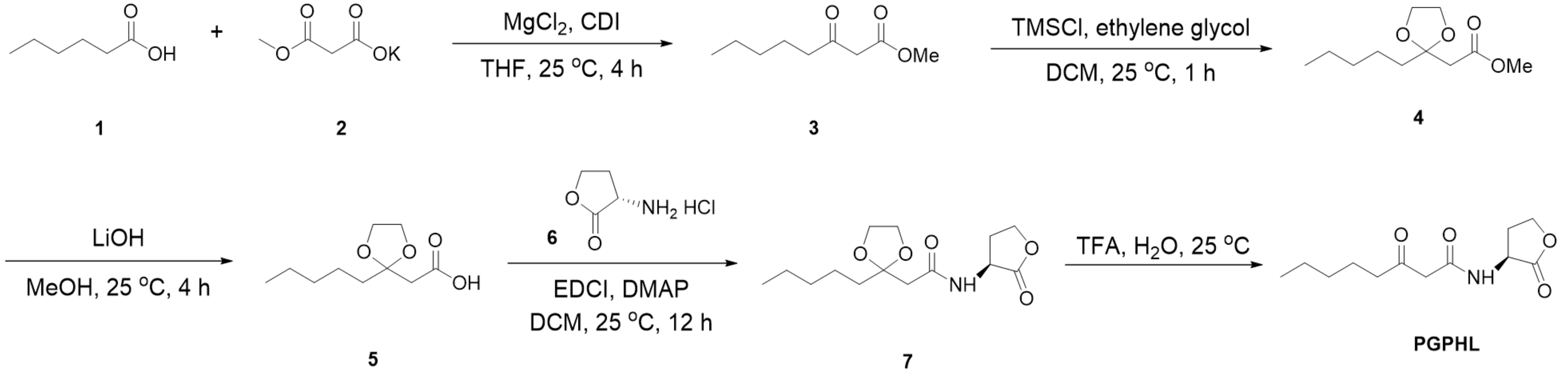

Fig. S9 Schematic diagram of PGPHL synthesis.

A

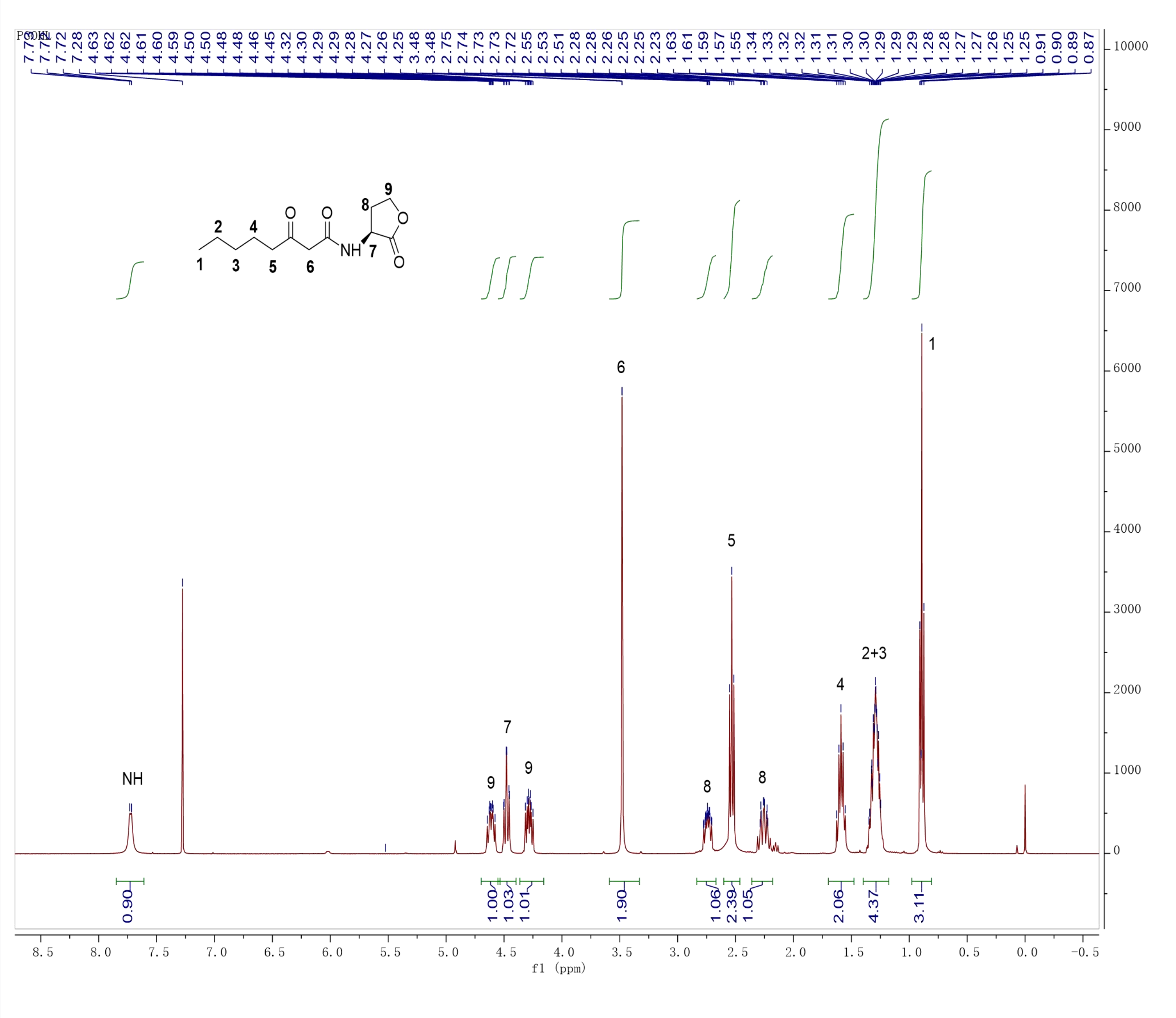

B

SAMPLE INFORMATION

Sample Name:	HR-1	Acquired By:	System
Sample Type:	Unknown	Sample Set Name:	0805
Vial:	110	Acq. Method Set:	ELSD 01
Injection #:	1	Processing Method:	14
Injection Volume:	3.00 ul	Channel Name:	ELSD Signal
Run Time:	12.0 Minutes	Proc. Chnl. Descr.:	ELSD Signal
Date Acquired:	8/5/2024 2:54:07 PM CST		
Date Processed:	8/6/2024 2:14:38 PM CST		

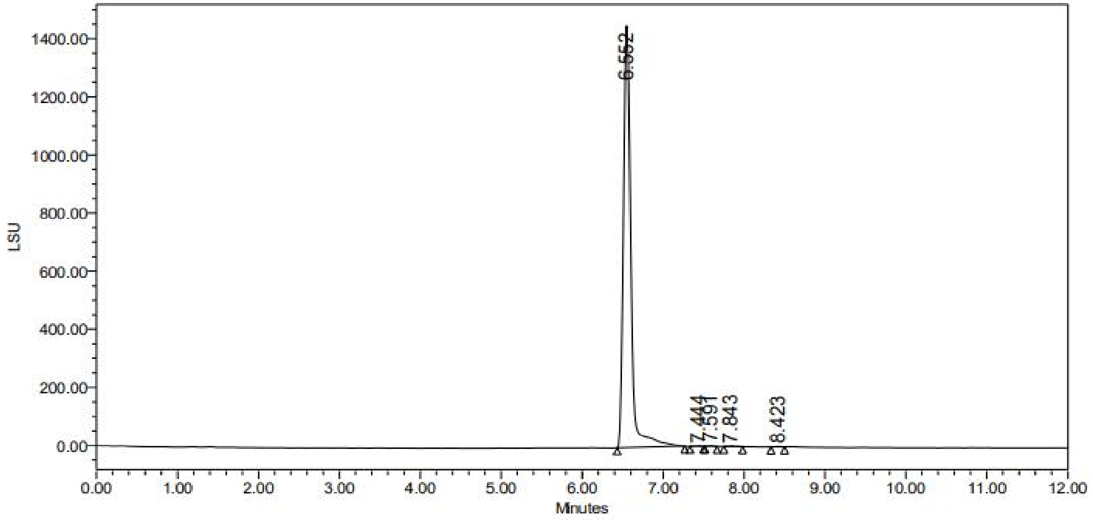

	RT	Name	Height	Area	% Area
1	6.552		1442154	9134688	99.68
2	7.444		646	4209	0.05
3	7.591		1789	7988	0.09
4	7.843		1395	10443	0.11
5	8.423		1115	6681	0.07

Fig. S10 Structural characterization and purity assessment of chemically synthesized PGPHL. The structure of PGPHL was confirmed by ^{1}H NMR (A), and its purity was analyzed by HPLC (B).
